# Chinese never smokers with adenocarcinoma of the lung are younger and have fewer lymph node metastases than smokers

**DOI:** 10.1186/s12931-022-02199-z

**Published:** 2022-10-29

**Authors:** Longyu Shan, Liang Zhang, Xiaolei Zhu, Zhilin Wang, Shaohan Fang, Junfeng Lin, Jianweng Wang, Ning Li, Hongming Liu, Xiaowen Zhang, Yihui Feng, Jingwei Liu, Jianyun Pan, Guanzhi Ye, Xiuyi Yu, Amanda Tufman, Alexander Katalinic, Torsten Goldmann, Frank Petersen, Jie Jiang, Guojun Geng, Xinhua Yu

**Affiliations:** 1 Department of Thoracic Surgery, Xiamen Cancer Hospital, The First Affiliated Hospital of Xiamen University, 361003 Xiamen, People’s Republic of China; 2 Priority Area Chronic Lung Diseases, Research Center Borstel, Member of the German Center for Lung Research (DZL), 23845 Borstel, Germany; 3 Borstel, Germany; 4 Pathology, Research Center Borstel, Member of the German Center for Lung Research (DZL), Borstel, Germany; 5 Borstel, Germany; 6 Department of Medicine V, Member of the German Center for Lung Research, University Hospital, LMU Munich, Munich, Germany; 7 Institute for Social Medicine and Epidemiology, University of Luebeck, Luebeck, Germany; 8 Teaching Hospital of Fujian Medical University, 361003 Xiamen, China

## Abstract

**Background:**

Lung cancers arising in never smokers have been suggested to be substantially different from lung cancers in smokers at an epidemiological, genetic and molecular level. Focusing on non-small cell lung cancer (NSCLC), we characterized lung cancer patients in China looking for demographic and clinical differences between the smoking and never-smoking subgroups.

**Methods:**

In total, 891 patients with NSCLC, including 841 with adenocarcinoma and 50 with squamous cell carcinoma, were recruited in this study. Association of smoking status with demographic and clinical features of NSCLC was determined, and risk factors for lymph node metastasis and TNM stage were evaluated using Multivariate logistic regression analysis.

**Results:**

In patients with adenocarcinoma, never smokers showed a younger age at diagnosis (54.2 ± 12.7vs. 59.3 ± 9.4, *p*^*adjusted*^<0.001), a lower risk for lymph node metastasis than smokers (7,6% vs. 19.5%, *p*^*adjusted*^<0.001) and less severe disease as indicated by lower percentages of patients with TNM stage of III or IV (5.5% vs. 14.7%, *p*^*adjusted*^<0.001 ). By contrast, these associations were not observed in 50 patients with squamous cell carcinoma. Multivariate logistic regression analysis showed that smoking status was a risk factor for lymph node metastasis (OR = 2.70, 95% CI: 1.39–5.31, *p* = 0.004) but not for TNM stage (OR = 1.18, 95% CI: 0.09–14.43, *p* = 0.896) in adenocarcinoma.

**Conclusion:**

This study demonstrates that lung adenocarcinoma in never smokers significantly differ from those in smokers regarding both age at diagnosis and risk of lymph node metastasis, supporting the notion that they are distinct entries with different etiology and pathogenesis.

**Supplementary Information:**

The online version contains supplementary material available at 10.1186/s12931-022-02199-z.

## Introduction

By far, lung cancer is the leading cause of cancer deaths worldwide making up 18% of all cancer deaths [1]. Despite cigarette smoking being the major risk factor for lung cancer, the degree of its association with different histological types varies considerably. The association is much stronger in squamous cell carcinoma (SQC) and small cell lung cancer (SCLC) than in adenocarcinoma (ADC) [2]. Consistently, smoking cession results a higher risk-reduction in SQC and SCLC than in ADC [3]. Epidemiological studies have shown that rates of lung cancers arising in never smokers have increased during recent decades [4–6]. As compared with lung cancers arising in smokers, those arising in never smokers show substantial differences at epidemiological, genetic and molecular levels, implying that they might be biologically distinct entities [7–10]. There is also a growing body of evidence showing differences in metastatic patterns in tumors harboring driver mutations [11]. In comparison, little is known about patterns of locoregional spread in early stage ADC in smokers vs. never smokers. As screening for lung cancer in some non-smoking persons has started to show promise [12], and targeted adjuvant therapies are becoming available, the clinical relevance of these differences will increase rapidly over the coming years.

Interestingly, the proportion of never smokers in patients with non-small cell lung cancer (NSCLC) varies highly across populations. For example, proportions of never smokers in patients with ADC in France and UK are less than 5% [13–14] , while these values in Japan and China are reportedly higher than 40% [15–17]. The high proportion of never smokers in patients in East Asia populations provides an opportunity to compare never smokers and smokers in NSCLC. In this study, we compared smoking and never-smoking patients with lung NSCLC and investigated differences in their demographical and clinical characteristics.

## Methods

### Patients

All patients with NSCLC in this study were recruited from the Department of Thoracic Surgery of the First Affiliated Hospital of Xiamen University, Xiamen, China from 2015 to 2021. Diagnosis of lung cancer was based on histology according to the 2015 World Health Organization Classification of Lung Tumors [18]. Patients were selected for lung cancer surgery according to the Chinese guidelines for diagnosis and treatment of primary lung cancer [19]. Briefly, the absolute indication of lung cancer surgery is the T1 − 3_N0 − 1_M0 disease, the relative indication of the surgery is part of T4_N0 − 1_M0 disease, the controversial indication is the T1 − 3_N2_M0 disease, and exploratory surgical indications for lung cancer include part of stage T1 − 3_N0 − 1_M1 with solitary metastases. All patients underwent segmentectomy or lobectomy with lymph node resection within two weeks after the diagnosis based on imaging and/or histopathological examination, and only patients with well-characterized demographic, clinical and pathological information as well as smoking status were recruited. Demographical, clinical and pathological information, including age at diagnosis, gender, smoking status, histological type, size of primary tumor, lymph node metastases and metastasis to distant organs were retrospectively collected from electronic medical records. To avoid the effect of treatment on the clinical features, patients receiving chemotherapy, radiotherapy, or biological therapies before surgery were excluded for the analysis. This study was performed in accordance with the 1964 Helsinki Declaration and its later amendments or comparable ethical standards, and the approval was obtained from the Ethic of Committees of the First Affiliated Hospital of Xiamen University. Since this retrospective study contains anonymous patient information, informed consent was waived by the institutional review board (IRB).

### Statistical analyses

All statistical analyses were performed with R software (R, version 4.1.1). For quantitative variables, data were shown as the mean ± SD (standard deviation). The Kolmogorov-Smirnov normality test or Shapiro-Wilk test were performed to examine if variables are normally distributed. To determine statistical significance, quantitative data in normal distribution were compared using the student t-test, otherwise the Mann-Whitney U test was used. For the categorical variables, data were expressed as number (percentage), and *p* values were calculated with Chi-Square test or Fisher’s exact test. *P* values were adjusted by Benjamini-Hochberg (BH) method for multiple comparisons and a *p* value less than 0.05 was considered as statistically significant. In addition, multivariate logistic regression (method ENTER) analysis was used to examine the influence of categorical variables such as smoking status (ever smoker/never smoker) and gender (male/female) as well as numeric variables including age on lymph node metastasis (LNMets) and TNM stages. For the multivariate logistic regression analysis, stages of primary tumor size, LNMets and metastasis were regarded as numeric variables. Odds ratios (OR), 95% CIs and *p* values were calculated for each variable.

## Results

### Clinical and demographic characteristics of patients

In total, 891 patients with NSCLC were recruited, including 841 with ADC and 50 with SQC (Table1). Mean age of patients with ADC was 55.2 ± 12.3 years (mean ± SD), and mean age of patients with SQC was 63.1 ± 6.8 years). Patients with ADC patients consisted of more females (58.3%) than males, while SQC patients were predominantly males (92%). As expected, majority of patients with SQC were smokers (68%), including current smokers and former smokers. By contrast, only 17% patients with ADC smoked. Proportion of never smokers in patients with ADC (83%) was approximately 2.6 times as high as that in patients with SQC (32%). Among the 891 patients with NSCLC, 66 were diagnosed with carcinoma in situ, and they were all ADC. When patients were categorized according to the size of primary tumor, the percentage of SQC patients with T2 to T4 was higher than that in ADC patients (61.9% vs. 16.6%). Also, the percentage of patients with lymph node metastasis (LNMets) in SQC was roughly 3 times as high as in ADC (28.6% vs. 9.6%), while no significant differences were observed regarding tissue metastasis between these two histological sub-types of NSCLC. With regards to TNM stages, the proportion of patients with severe disease (with a TNM stage III and IV) in SQC was higher than that in ADC (20.4% vs. 7.0%). (Table1)

**Table 1:** Demographic and clinical features of NSCLC patients

		NSCLC (n = 891)	
		**Adenocarcinoma (n = 841 )**	**Squamous-cell carcinoma (n = 50 )**
**Age at diagnosis**		55.2 ± 12.3	63.1 ± 6.8
**Sex**	male	351 (41.74%)	44 (88.00%)
	female	490 (58.26%)	6 (12.00%)
**Smokingstatus**	Never smoker	698 (83.00%)	16 (32.00%)
	Former smoker	5 (0.59%)	6 (12.00%)
	Current smoker	138 (16.41%)	28 (56.00%)
**T**	Tis	66 (8.06%)	0 (0%)
	T1a	256 (31.26%)	2 (4.08%)
	T1b	264 (32.23%)	7 (14.29%)
	T1c	97 (11.84%)	10 (20.41%)
	T2a	86 (10.50%)	12 (24.49%)
	T2b	14 (1.71%)	6 (12.24%)
	T3	32 (3.91%)	7 (14.29%)
	T4	4 (0.49%)	5 (10.02%)
**N**	N0*	760 (90.37%)	35 (71.43%)
	N1	37 (4.40%)	9 (18.37%)
	N2	43 (5.11%)	5 (10.20%)
	N3	1 (0.12%)	0 (0%)
**M**	M0	830 (98.69%)	49 (100%)
	M1	11 (1.31%)	0 (0%)
**TNM**	0	65 (7.85%)	0 (0%)
	IA1	253 (30.56%)	2 (4.08%)
	IA2	241 (29.11%)	6 (12.24%)
	IA3	90 (10.87%)	7 (14.29%)
	IB	73 (8.82%)	9 (18.37%)
	IIA	9 (1.09%)	4 (8.16%)
	IIB	39 (4.71%)	11 (22.45%)
	IIIA	45 (5.43%)	8 (16.33%)
	IIIB	4 (0.48%)	1 (2.04%)
	IVA	9 (1.09%)	1(2.04%)

^#^22 ADC patients (9 smokers and 13 never smokers) with T1 or T2 stage and 5 ADC patients with IA stage had no information of substages. One squamous-cell carcinoma patient is missingOne SQC patient in T2 stage had no information of substage, and 1 SQC patient had no information of N, M and TNM

### Association of smoking status with characteristics of ADC

We first examined the association of smoking status with demographical and clinical characteristics of patients with ADC. Among 841 patients with ADC, 698 were never smokers and 143 were ever smokers, including 5 former smokers and 138 current smokers. Notably, the mean age at diagnosis of never smokers was approximately 5 years younger than that of ever smokers (54.2 ± 12.6vs. 59.3 ± 9.4, *p*^*adjusted*^<0.001) (Table2 and supplementary Fig.1A). Significant association of smoking status with gender was also observed. In smokers 91.2% were male, while in non-smokers only 31.2% were male (*p*^*adjusted*^<0.001). Percentages of ADC in situ were comparable between smokers and never smokers, and no significant difference was observed in size of primary tumors between the two subgroups. However, significant a difference was observed regarding LNMets; the proportion of patients showing LNMets was approximately 2.5 times in smokers than in never smokers (19.6% vs. 7.6%, *p*^*adjusted*^<0.001). In addition, smokers showed a higher percentage of patients with severe disease as defined by TNM stages of III and IV than never smokers (14.7% vs. 5.49%, *p*^*adjusted*^<0.001). (Table2)

**Table 2:** Association of smoking status with demographical and clinical features of patients with adenocarcinoma

			Ever Smoker (n = 143)		Never smoker (n = 698 )		P values	P adj
**Age at diagnosis**			59.3 ± 9.4		54.2 ± 12.7		0.0000015	< 0.001
**Sex**	male		135 (94.41%)		216 (30.95%)		1.18e-44	< 0.001
	female		8 (5.59%)		482 (69.05%)			
**T**	Tis		3 (2.24%)		63 (9.20%)			
	T1a	T1*	26 (19.40%)	100 (74.63%)	230 (33.58%)	517 (75.47%)	0.067	Ns
	T1b		56 (41.79%)		208 (30.36%)			
	T1c		18 (13.43%)		79 (11.53%)			
	T2a	T2-T4	20 (14.93%)	31 (23.13%)	66 (9.64%)	105 (15.33%)		
	T2b		5 (3.73%)		9 (1.31%)			
	T3		5 (3.73%)		27 (3.94%)			
	T4		1 (0.75%)		3 (0.44%)			
**N**	N0*		115 (80.42%)		645 (92.41%)		0.000014	< 0.001
	N1-N3		28 (19.48%)		53 (7.59%)			
**M**	M0*		141 (98.60%)		689 (98.71%)		1	Ns
	M1		2 (1.40%)		9 (1.29%)			
**TNM**	0		3 (2.21%)		62 (8.96%)			
	IA1	Less severe *	25 (18.38%)	113 (83.09%)	228 (32.95%)	592 (85.55%)	0.00037	< 0.001
	IA2		45 (33.09%)		196 (28.32%)			
	IA3		16 (11.76%)		74 (10.69%)			
	IB		15 (11.03%)		58 (8.38%)			
	IIA		3 (2.21%)		6 (0.87%)			
	IIB		9 (6.62%)		30 (4.34%)			
	IIIA	Severe	17 (12.50%)	20 (14.71%)	28 (4.05%)	38 (5.49%)		
	IIIB		1 (0.74%)		3 (0.43%)			
	IV		2 (1.47%)		7 (1.01%)			

*reference group for the comparison of T, N, M and TNM. 22 patients (9 smokers and 13 never smokers) with T1 or T2 stage had no information of substages. 5 patients with IA stage had no information of substages

### Association of smoking status with characteristics of SQC

Having demonstrated the association of smoking status with age at diagnosis, gender, LNMets and TNM stage, we sought to investigate whether the association is ADC specific. For this purpose, we examined the effect of smoking status on SQC. The 50 patients with SQC consisted of 34 smokers and 16 never smokers. In contrast to ADC, age at diagnosis in SQC patients who never smoked was older, although not significant after adjustment for multiple comparison, than that in patients who were smokers (66.0 ± 7.1 *vs* 61.7 ± 6.3, *p* = 0.035) (Table3 and supplementary Fig.1B). Similar to ADC, SQC was also characterized by a significantly higher male-to-female ratio in ever smokers than in never smokers (*p*^*adjusted*^=0.003). Regarding LNMets, 29.4% of SQC patients who were smokers showed lymph node spreading, which was not significantly different to the prevalence of lymph node involvement (26.67%) in never smokers. In addition, no significant difference was observed in disease severity as indicated by TNM stages between the two groups. 

**Table 3:** Association of smoking status with demographical and clinical features of patients with squamous-cell carcinoma

			Ever Smoker (n = 34)		Never smoker (n = 16 )		P values	P adj
**Age at diagnosis**			61.7 ± 6.33		66.0 ± 7.1		0.035	Ns
**Sex**	male		34 (100.00%)		10 (62.50%)		0.0005	0.003
	female		0 (0%)		6 (37.50%)			
**T**	Tis		0 (0%)		0 (0%)			
	T1a	T1*	0 (0%)	12 (35.29%)	2 (13.33%)	7 (46.67%)	0.451	Ns
	T1b		4 (11.76%)		3 (20.00%)			
	T1c		8 (23.53%)		2 (13.33)			
	T2a	T2-T4	8 (23.53%)	22 (64.71%)	4 (26.67%)	8 (53.33%)		
	T2b		4 (11.76%)		2 (13.33%)			
	T3		7 (20.59%)		0 (0%)			
	T4		3 (8.82%)		2 (13.33%)			
**N**	N0*		24 (70.59%)		11 (73.33%)		1	Ns
	N1-N3		10 (29.41%)		4 (26.67%)			
**M**	M0*		34 (100%)		15 (100%)		1	Ns
	M1		0 (0%)		0 (0%)			
**TNM**	0		0 (0%)		0 (0%)			
	IA1	Less severe *	0 (0%)	28 (82.35%)	2 (13.33%)	11 (73.33%)	0.470	Ns
	IA2		4 (11.76%)		2 (13.33%)			
	IA3		6 (17.65%)		1 (6.67%)			
	IB		6 (17.65%)		3 (20.00%)			
	IIA		2 (5.88%)		2 (13.33%)			
	IIB		10 (29.41%)		1 (6.67%)			
	IIIA	Severe	5 (14.71%)	6 (17.65%)	3 (20.00%)	4 (26.67%)		
	IIIB		1 (2.94%)		0 (0%)			
	IV		0 (0%)		1 (6.67%)			

*reference group for the comparison of T, N, M and TNM. One patient in T2 stage had no information of substage. One patient had no information of N, M and TNM.

### Risk factors for LNMets and severe disease

Given the above results showed that smoking status was associated with LNMmet and TNM stage in ADC, we next investigated whether cigarette smoking is an independent risk factor for the two clinical characteristics using multivariate logistic regression analysis. As shown in Table4, size of primary tumor was the strongest factor associated with the risk of LNMets, where a 1-stage increase in tumor grade was associated with a roughly 3 times higher risk for LNMets (OR = 3.81, 95% CI: 2.74-5,34, *p* < 0.001). Besides size of primary tumor, smoking status was another risk factor for LNMets. Smokers showed an increased risk for LNMets, with an OR value of 2.70 (95% CI: 1.39–5.31, *p* = 0.004). By contrast, LNMets was not significantly associated with age and gender of patients. As expected, primary tumor size, LNMets and metastasis were the main independent risk factors for severe disease (TNM stage III or IV). In addition, age at diagnosis was identified to be a risk factor for severe disease (OR = 0.92, 95% CI: 0.84–0.99, *p* = 0.04). Although smoking status was significantly associated with severe disease, the multivariate logistic regression analysis showed it was not an independent risk factor (OR = 1.18, 95% CI: 0.09–14.43, *p* = 0.896). (Table5)

**Table 4:** Logistic regression analysis of lymph node metastasis in adenocarcinoma patients

Independent variables	***OR***	95% CI of ***OR***		P value
		**Upper**	**Lower**	
Smoking Status	2.70	5.31	1.39	0.0035
Age at diagnosis	1.02	1.04	0.99	0.07
Tumor size	3.81*	5.34	2.74	2.3 × 10^− 15^
Gender(Male)	0.94	1.73	0.50	0.9750

*tumor size was regarded as a numeric variable, OR was calculated for each 1-stage increase in tumor size

**Table 5:** Logistic regression analysis of TNM in adenocacinoma patients

Independent variables	***OR***	95% CI of ***OR***		P value
		**Upper**	**Lower**	
Smoking Status	1.18	14.43	0.09	0.896
Age at diagnosis	0.92	0.99	0.84	0.040
Gender(Male)	1.41	9.80	0.22	0.72
Tumor size	17.51*	99.51	5.36	< 0.001
LNMets	265.40	2857.80	60.10	< 0.001
Metastasis	1.87 × 10^11^	NA	1.15 × 10^− 68^	0.99

*Tumor size, LNMets and Metastasiswere regarded as numeric variables, OR was calculated for each 1-stage increase in tumor size

## Discussion

In the present study, we investigated the role of smoking status, age, and gender in a clinical cohort of patients with NSCLC who underwent thoracic surgery. Since it has been shown that CT Screening promotion is associated with lung cancer overdiagnosis among Asian women [20], precise staging is a strength of the surgical cohorts in the current study. We demonstrate that ever smokers show a higher age at diagnosis, a higher male-to-female ratio, an increased risk of LNMets and more severe disease than never smokers in patients with ADC. Moreover, multiple multivariate logistic regression analysis shows that smoking is an independent risk factor for LNMets.

Notably, 83% of patients with lung ADC in the present study are never smokers. This finding is line with the observation that proportion of never smokers in ADC patients is much higher than in East Asian than in Europe [21–22] . The difference between East Asia and Europe suggests that population-specific genetic and environmental factors contribute to the development of ADC in never smokers [23]. In line with this notion, genetic studies have shown that lung ADC arising in never smokers is associated with different genetic variants in Asian populations compared to Caucasian populations [24]. Besides genetics factor, multiple environmental risk factors contributing to the development of COPD vary considerably cross the world[25] .

As expected, most smokers with ADC are males, while roughly two-thirds of never smokers with ADC are females. This result is in consistent with a previous population-based study in Japan, where Seki and colleagues reported that percentages of male in ever smokers and never smokers in patients with lung ADC are 87% and 16%, respectively [26]. The male predominance in ever smokers can only be explained in part by an extremely low female smoking prevalence in China. According to a previous study, only 3.2% are ever regular smokers among 302 669 women enrolled in 2004–08, while the male smoking prevalence in 210 222 men enrolled in the same time period is approximately 70% in China [27]. The female predominance in never smokers in ADC might be due to at least two factors. The dramatic difference in smoking prevalence between men and women leads to a high female-to-male ratio in never smokers in general population, which partially accounts for the predominance of female in lung ADC patients arising in never smokers. In addition, the female predominance might also be due, in part, to the higher risk of lung cancer in women than in men, a hypothesis supported by epidemiological evidence [28]. A comprehensive molecular characterization for instance of by determining EGFR, K-RAS and other mutations by sequencing, droplet-PCR or mutation-specific antibodies w has been completed in similar contexts in other cohorts and suggests differences in the underlying molecular events [29–31].

Interestingly, although the smoking status is associated with both with LNMets and TNM stages in ADC, multivariate logistic regression analysis demonstrates that cigarette smoking is only an independent risk factor for LNMets, with an OR of 2.70. This finding suggests that the association of smoking status with TNM stages is due to the contribution of cigarette smoking to LNMets. In a previous multicenter retrospective study, Chen et al. examined risk factors for LNMets in 10 885 patients with clinical T1 NSCLC [32]. Their analyses demonstrated that smokers showed a significant higher prevalence of LNMets than never smokers (22.8% vs. 13.6%, *p* < 0.001), while the multivariate analyses failed to show smoking status as a significant risk factors for LNMets [33]. A possible explanation for the discrepancy between our results and the findings from Chen‘s study could be the difference in types of NSCLC investigated. In the present study, we examined the impact of smoking status on LNMets in ADC and SQC separately, while Chen and colleagues combined these two entities for the analysis. As shown in the current study, significant association of cigarette smoking with an increased risk of LNMets was only observed in ADC, but not in SQC. Therefore, combination of ADC and SQC likely reduces the statistical power of the analysis. Besides LNMets, age at diagnosis is also associated with cigarette smoking in both the discovery and replication cohorts in the present study, where never smokers are roughly 5 years younger than smokers. This difference was also observed by Lam and colleagues who reported that median ages of never smokers and smokers in 115 patients with lung ADC are 54 and 60 years, respectively, although the difference is not statistically significant due to the small sample size [34].

Given that LNMets develop during cancer progression [35], it is conceivable to speculate that smokers might have symptomatic comorbid illnesses which might lead to a delayed lung cancer diagnosis and consequent high prevalence of LNMets in lung ADC. However, this speculation is contradicted by two findings from the present study. First, the primary tumor size was comparable between smokers and never smokers in ADC, indicating no evidence of a delay of diagnosis in ever smokers compared to never smokers. Second, never smokers and smokers in patients with SQC showed comparable prevalence of LNMets, suggesting that the association of cigarette smoking with LNMets might be ADC-specific. However, due to the sample size of the SQC cohort in the current study, the association of smoking status with LNMets in SQC needs to be further elucidated. A possible explanation for the association of smoking status with age at diagnosis and the risk of LNMets is that lung ADC arising in never smokers differs from that arising in smokers in disease etiology and pathogenesis. This notion is supported by the evidence that prevalence of oncogenic driver mutations in lung ADC in never smokers is significantly higher than that in smokers [36–37]. Until very recently molecular testing was not clinically indicated in early stage, surgically treated NSCLC, and so this data is not available for our cohorts. However, other data sets have reliably described clinically relevant differences in this context and further studies are planned to characterise the available tissue samples, with a focus on both molecular driver alterations and differences in immune response.

LNMets are initiated by the migration of cancer cells into lymphatic capillaries, and the development of LNMets likely depends on a combination of intrinsic properties of cancer cells and microenvironment of LN [38–39]. Cigarette smoke is a complex mixture of numerous chemicals including multiple lung carcinogens [40]. Nicotine, a component responsible for tobacco addiction, also plays a role in the invasion and metastasis of various cancers, including lung cancer [41]. Using a mouse model of lymphatic metastasis, Shimizu et al. demonstrated that nicotine promotes LNMets in head and neck squamous cell carcinoma by inducing nuclear accumulation of phosphorylated epidermal growth factor (pEGFR) and activation of Akt signaling in neck squamous cell carcinoma cells [42]. Therefore, it is intriguing to examine whether the nicotine induced pEGFR-Akt signaling pathway also contributes to the risk effect of cigarette smoking mediated LNMets in lung ADC.

A major limitation of the current study is the lack of information of secondhand smoke in never smokers. For this reason, it is not possible to determine the effect of passive smoking in the development of lung cancer. According to a systematic meta-analysis performed by Du et al., approximately 16% of lung cancer cases among never smokers in China are potentially attributable to passive smoking [43]. Therefore the potential effect of passive smoking in the development of lung ADC in never smoker needs to be taken account when interpreting this study.

## Conclusion

The present study demonstrates that lung ADC in never smokers differs significantly to that in smokers in age at diagnosis and the risk for LNMets, supporting the notion that they are distinct entries with different etiology and pathogenesis. Furthermore, this study also indicates that cigarette smoking could be an independent risk factor for LNMets in lung ADC. The predisposition of smoking-related ADC to spread to regional lymph nodes may indicate differences in locoregional antitumoral immune response in smokers vs. never smokers, which should be the focus of further investigation.

## Electronic supplementary material

Below is the link to the electronic supplementary material.

Supplementary Material 1

## Data Availability

All data generated or analysed during this study are included in this published article and are available from the corresponding author on reasonable request.

## Abbreviations

NSCLC: non-small cell lung cancer.
ADC: adenocarcinoma.
SQC: squamous cell carcinoma.
NS: never smokers.
FS: former smokers.
CS: current smokers.
LNMets: lymph node metastasis.
