# Supplementary material for: Chinese never smokers with adenocarcinoma of the lung are younger and have fewer lymph node metastases than smokers
Source: Respir Res. 2022 Oct 29;23:293. doi: 10.1186/s12931-022-02199-z (PMC9617301; doi:10.1186/s12931-022-02199-z)
Supplement: Supplementary file 1 — Supplementary Material 1 [file 12931_2022_2199_MOESM1_ESM.pdf]

Supplementary information

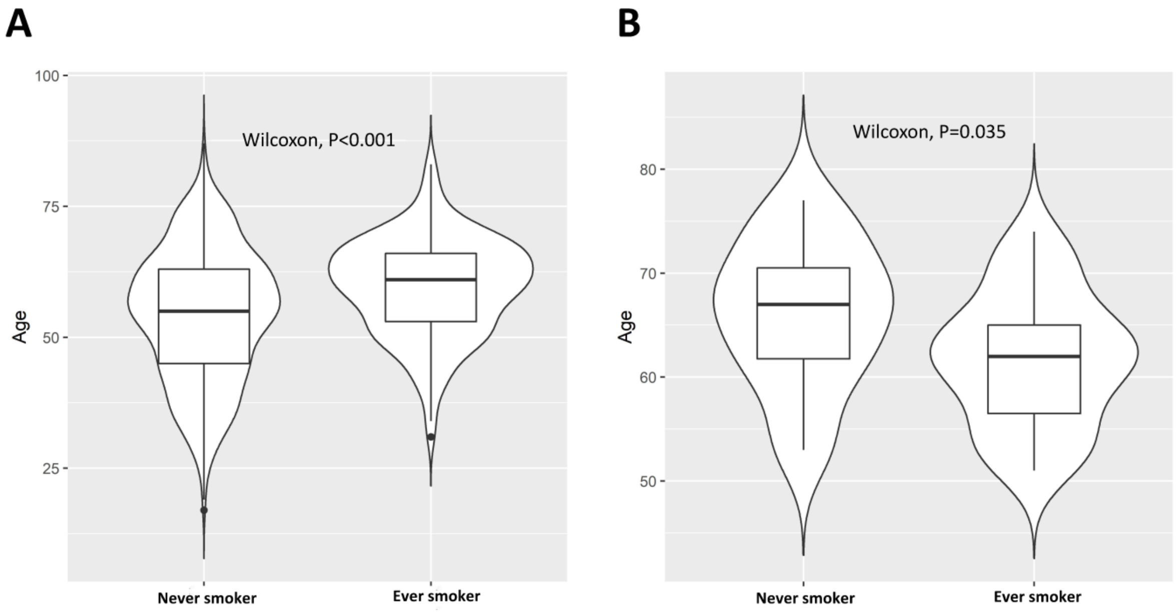

Supplementary figure 1. Violin plot of age at diagnosis in patients with NSCLC. Patients with adenocarcinoma (A) or squamous cell carcinomas (B) were divided into two subgroups according to smoking statues, namely never smokers and ever smokers. The median and interquartile range of age at diagnosis are shown as bold horizontal line and box, respectively. Statistical difference in age at diagnosis between smokers and never smokers was determined by using Wilcoxon signed rank test.
